# Supplementary material for: Mapping the evolution of fertility support policies in China: A content and instrumental analysis
Source: PLoS One. 2025 Oct 9;20(10):e0332137. doi: 10.1371/journal.pone.0332137 (PMC12510515; doi:10.1371/journal.pone.0332137)
Supplement: S1 Appendix — (ZIP) [file pone.0332137.s001.zip › S1 Appendix. 226 original policy documents/85-国务院关于印发中国妇女发展纲要和中国儿童发展纲要的通知(FBM-CLI-2-292888).doc]

国务院关于印发中国妇女发展纲要和中国儿童发展纲要的通知

**发布部门：** [国务院](https://www.pkulaw.com/law/chl?Aggs.IssueDepartment=5)

**发文字号：**国发〔2001〕18号

**发布日期：**2001.05.22

**实施日期：**2001.05.22

**时效性：** [失效](https://www.pkulaw.com/law/chl?Aggs.TimelinessDic=02)

**效力级别：** [国务院规范性文件](https://www.pkulaw.com/law/chl?Aggs.EffectivenessDic=XC0203)

**法规类别：** [老少妇幼残保护](https://www.pkulaw.com/law/chl?Aggs.Category=01604)

**失效依据：**[国务院关于宣布失效一批国务院文件的决定](javascript:TMP(267463,0,'chl'))

国务院关于印发中国妇女发展纲要和中国儿童发展纲要的通知
（国发〔2001〕18号）

各省、自治区、直辖市人民政府，国务院各部委、各直属机构：
　　《[中国妇女发展纲要（2001-2010年）](https://www.pkulaw.com/chl/37733dd7246afab4bdfb.html)》和《中国儿童发展纲要（2001-2010年）》已经国务院同意，现印发给你们，请认真贯彻执行。

国务院
二○○一年五月二十二日

[中国妇女发展纲要](https://www.pkulaw.com/chl/37733dd7246afab4bdfb.html)
（2001-2010年）

　　前言

　　随着新世纪钟声的敲响，中国妇女运动和妇女事业又一次站在新的历史起点，开始新的伟大进军。
　　妇女发展作为全球经济和社会发展的重要组成部分，受到国际社会的普遍重视。在过去的几十年里，国际社会为促进妇女发展与进步达成了多项协议，将妇女问题与全球政治、经济发展紧密相联成为国际社会的共识，消除经济全球化进程对妇女产生的不利影响正逐步纳入各国政府的重要议程。我国政府始终把维护妇女权益、促进妇女发展作为义不容辞的责任。1995年制定和发布的《[中国妇女发展纲要（1995-2000年）](https://www.pkulaw.com/chl/33670a50094f0c40bdfb.html)》（以下简称[’95《纲要》](https://www.pkulaw.com/chl/33670a50094f0c40bdfb.html)）是我国妇女发展的重要里程碑。5年多来，在国务院和地方各级政府的积极努力下，在包括非政府组织在内的社会力量的大力支持下，[’95《纲要》](https://www.pkulaw.com/chl/33670a50094f0c40bdfb.html)的主要目标基本实现，为21世纪的妇女发展奠定了良好的基础。[’95《纲要》](https://www.pkulaw.com/chl/33670a50094f0c40bdfb.html)的实施改善了我国妇女生存与发展的社会环境，维护了妇女的合法权益，加速了男女平等的进程，妇女在政治、经济、教育、健康等各个领域取得了全面进步。
　　2001-2010年，是我国经济和社会发展的重要时期，也是完善社会主义市场经济体制和扩大对外开放的重要时期。我国将在更广泛的领域和更高的层次参与经济全球化。面对我国改革开放和现代化建设的新形势、新任务，面对经济全球化的发展趋势，我国的妇女发展必须有更高的目标和更快的前进步伐。
　　为了更好地维护妇女权益，提高妇女整体素质，加快实现男女平等的进程，发挥广大妇女在社会主义现代化建设中的重要作用，我国政府制定并发布《[中国妇女发展纲要（2001-2010年）](https://www.pkulaw.com/chl/37733dd7246afab4bdfb.html)》（以下简称[《纲要》](https://www.pkulaw.com/chl/37733dd7246afab4bdfb.html)）。[《纲要》](https://www.pkulaw.com/chl/37733dd7246afab4bdfb.html)根据《[中华人民共和国国民经济和社会发展第十个五年计划纲要](https://www.pkulaw.com/chl/f439200c548488c1bdfb.html)》的总体要求，从我国基本国情和妇女现状出发，兼顾妇女发展的阶段性目标和长远目标，确定了2001-2010年妇女发展的总目标和主要目标。同时，充分考虑第四次世界妇女大会《行动纲领》提出的妇女发展12个重要领域，借鉴世界上其他国家制定妇女发展规划的做法，以[’95《纲要》](https://www.pkulaw.com/chl/33670a50094f0c40bdfb.html)的实施成效为基础，根据我国妇女发展迫切需要解决的实际问题和2001-2010年的可持续发展，[《纲要》](https://www.pkulaw.com/chl/37733dd7246afab4bdfb.html)确定了6个优先发展领域，即：妇女与经济、妇女参与决策和管理、妇女与教育、妇女与健康、妇女与法律、妇女与环境，并把促进妇女发展的主题贯穿始终。
　　[《纲要》](https://www.pkulaw.com/chl/37733dd7246afab4bdfb.html)的制定和实施，目的是强化政府的有关职能，动员全社会的力量，为妇女的进步与发展创造更好的社会环境。同时，鼓励妇女在参与经济和社会发展的过程中争取自身的进步与发展。
　　21世纪，中国妇女事业必将迎来一个更加美好的春天。

　　总目标

　　贯彻男女平等的基本国策，推动妇女充分参与经济和社会发展，使男女平等在政治、经济、文化、社会和家庭生活等领域进一步得到实现。保障妇女获得平等的就业机会和分享经济资源的权利，提高妇女的经济地位；保障妇女的各项政治权利，提高妇女参与国家和社会事务管理及决策的水平；保障妇女获得平等的受教育机会，普遍提高妇女受教育程度和终身教育水平；保障妇女享有基本的卫生保健服务，提高妇女的健康水平和预期寿命；保障妇女获得平等的法律保护，维护妇女的合法权益；优化妇女发展的社会环境和生态环境，提高妇女生活质量，促进妇女事业的持续发展。

　　主要目标与策略措施

　　**一、**妇女与经济
　　（一）主要目标。
　　1.保障妇女获得经济资源的平等权利和机会。
　　2.消除就业性别歧视，实现男女平等就业，保障妇女劳动权利，妇女从业人员占从业人员总数的比例保持在40%以上。
　　3.妇女享有与男子平等的社会保障权利。城镇职工生育保险覆盖面达到90%以上。
　　4.保障女职工享有特殊劳动保护。
　　5.缓解妇女贫困程度，减少贫困妇女数量。
　　（二）策略措施。
　　妇女与男子平等获得经济权利、共享经济资源和社会发展成果是妇女发展的基础条件。
　　1.国家宏观政策。
　　国家的经济和社会发展规划应体现妇女发展的主要目标，经济分析和经济结构调整应纳入性别平等观念。
　　--制定妇女平等参与经济发展的方针政策，提供妇女享有与男子平等的参与经济决策的机会和途径，缩小男女在分享经济决策权上的差距，提高妇女参与经济决策及管理的水平。
　　--确保妇女平等获得经济资源和有效服务。主要包括获得资本、信贷、土地、技术、信息等方面的权利；农村妇女享有与居住地男子平等的土地承包权、生产经营权、宅基地分配权、土地补偿费、股份分红等权利。
　　--改善妇女经济状况，进行适当的有利于妇女生存发展的专项投资。
　　2.法律和部门政策。
　　制定和完善有利于妇女平等参与经济和社会发展、平等就业等相关法律法规和政策。
　　--贯彻落实《[中华人民共和国劳动法](https://www.pkulaw.com/chl/15fe2d94afee49a2bdfb.html)》等相关法律法规，禁止招工、招聘中的性别歧视。
　　--保障妇女享有与男子平等参与资本、技术等生产要素的分配权。保障多元化分配形式中的男女同工同酬，同工种、同类别从业人员中女性工资与男性工资相同。缩小男女收入差距。
　　--拓宽妇女就业渠道，在经济发展和产业结构调整中，充分考虑妇女就业的需要，大力发展第三产业特别是社区服务业，为妇女创造新的就业机会和就业岗位。提高妇女在新兴产业、新兴行业中的就业比例和中、高级专业技术人员中的女性比例。提倡自主就业，鼓励妇女自谋职业，支持和引导妇女兴办私营、个体企业和发展科技型中小企业，促进妇女通过多种形式再就业。
　　--进一步落实女职工劳动保护政策，为女职工提供必要的工作和劳动条件，解决女职工在劳动和工作中因生理特点造成的特殊困难。
　　--指导各类用人单位把女职工特殊劳动保护条款纳入劳动合同和集体合同，健全和完善女职工特殊劳动保护措施，不断改善女职工劳动条件。加强对女职工特殊劳动保护法律法规和政策的宣传教育及培训，提高用人单位的法制意识和安全生产意识，提高女职工的自我保护意识。
　　--加大劳动保障监察执法力度，切实保障妇女的劳动权利。对于违反《[中华人民共和国劳动法](https://www.pkulaw.com/chl/15fe2d94afee49a2bdfb.html)》、《[中华人民共和国妇女权益保障法](https://www.pkulaw.com/chl/dff2eefbddf155b3bdfb.html)》、《[女职工劳动保护规定](https://www.pkulaw.com/chl/133408887a349cdabdfb.html)》等相关法律法规、侵犯妇女合法权益的行为，要依法处理。
　　--引导和扶持农村妇女富余劳动力向非农产业转移。面向农村妇女开展各种劳动技能培训，帮助其从传统种植业向非农产业转移；在城镇化建设过程中，有组织地开展劳务输出，为农村妇女在非农产业就业创造更多的机会。
　　--制定减少妇女贫困的政策措施，增加贫困妇女的经济收入。在实施西部大开发战略中，加大对贫困妇女的扶持力度，按照同等优先的原则，帮助、支持贫困妇女实施扶贫项目，鼓励、支持以妇女为主的扶贫经济实体的发展，使贫困妇女成为扶贫资源的获得者和扶贫成果的直接受益者。
　　3.社会保障和服务。
　　进一步完善社会保障体系，积极推动在不同所有制经济实体就业的、不同收入层次的妇女按照国家规定参加社会保险，确保妇女在参与经济发展中接受有效服务。
　　--妇女享有与男子平等参加城镇职工基本养老保险、基本医疗保险、失业保险、工伤保险、生育保险的权利。
　　--普遍建立城镇职工生育保险制度，完善相关配套措施，切实保障女职工生育期间的基本生活和医疗保健需求。
　　--为妇女劳动就业提供信息、服务和培训。加强公共职业介绍机构对妇女的职业指导、职业介绍等就业服务。开展有针对性的就业培训，提高妇女的就业能力。
　　--为残疾妇女提供就业服务和职业技能培训，提高残疾妇女的就业比例。
　　--鼓励和支持社会团体开展有利于提高妇女劳动生产技能的各项培训活动。

　　**二、**妇女参与决策和管理
　　（一）主要目标。
　　1.提高妇女参与国家和社会事务的管理及决策水平。
　　2.提高妇女参与行政管理的比例。各级政府领导班子中要有1名以上女干部。国家机关部（委）和省（自治区、直辖市）、地（市、州、盟）政府工作部门要有一半以上的领导班子配备女干部。正职或重要岗位女性数量要有较大的增加。
　　3.女干部占干部队伍总数的比例逐步提高。
　　4.女性较集中的部门、行业管理层中的女性比例与女职工比例相适应。
　　5.村民委员会、居民委员会成员中女性要占一定比例。
　　6.扩大妇女民主参与的渠道，提高妇女民主参与的水平。
　　（二）策略措施。
　　妇女广泛参与国家和社会事务管理、享有充分的民主和自由是国家文明进步的重要标志。
　　1.国家宏观政策。
　　在更大范围和更大程度上推进妇女参与国家和社会事务管理及决策的进程，扩大妇女民主参与的渠道。
　　--充分保障妇女参与国家和社会事务管理及决策的权利。引导妇女依法参与经济、文化和社会事务的管理，提高妇女参政议政的水平和比例。
　　--在干部人事制度改革中，完善平等竞争机制和公务员管理制度，健全监督检查机制，为妇女参与决策和管理提供平等的竞争机会。在同等条件下优先选拔女干部。
　　--在制定涉及妇女根本利益的方针政策时，要听取各级人大女代表、政协女委员以及妇女组织、妇女群众的意见和建议。
　　--充分发挥各级妇联组织的民主参与和民主监督作用，使其成为联系妇女群众的桥梁和纽带，成为反映妇女群众意见和建议的重要渠道。
　　--重视妇联组织在培养选拔女干部、妇女参政议政方面的意见和建议。
　　2.法律和部门政策。
　　制定和完善有利于妇女平等参与决策和管理的相关法律法规和政策，鼓励、引导妇女积极参与竞争和民主管理。
　　--按照有关部门制定的培养、选拔女干部规划，实现省（自治区、直辖市）、地（市、州、盟）和县（市、区、旗）领导班子中女干部配备目标。
　　--政府所属部门领导层中女干部数量逐步增加。逐步提高女性较集中的部门、行业管理层中的女性比例，教育、科技、文化、卫生、体育、计划生育、民政、司法、劳动和社会保障等部门领导班子中要首先配备女干部。
　　--干部的选拔、聘用、晋升及公务员的录用，要切实贯彻“公开、公平、竞争、择优”的原则，保障女性不受歧视。
　　--加强对年轻优秀女干部的培养并优先选拔。各级领导班子后备干部队伍中应有一定数量的女干部。
　　--提高女干部的政治文化素质，大学本科以上学历的女干部应有较大幅度的增加。
　　--企业事业单位招聘专业技术和管理人员，不得以性别为由拒绝录用符合条件的女性，在聘用合同中不得含有性别歧视的内容。
　　--结合建立现代企业制度的实践，注意培养和发现高层次的女性管理人才。国有企业要积极探索在企业董事会、监事会、经理等决策、管理层发挥妇女民主参与的新形式，提高企业领导班子成员中的女性比例。
　　--坚持和完善以职工代表大会为基本形式的企业民主管理制度，企业职工代表大会中女代表比例应与女职工比例相适应。
　　--在民主选举过程中，鼓励妇女行使选举权和被选举权，积极参与选举，提高妇女民主参与的程度和比例。
　　--扩大基层民主，鼓励和推动妇女参与讨论和决定基层公共事务，保障妇女直接行使民主权利。妇女代表会主任作为候选人提名，经选举进入居民委员会和村民委员会。村民代表大会中女代表的比例要有较大幅度的提高。
　　3.社会宣传和培训。
　　提高全社会对妇女参与决策和社会事务管理重要性的认识，为妇女参政议政创造良好的社会环境。保障妇女享有与男子平等的培训教育机会。
　　--加强对女干部的培训教育和轮岗锻炼，培养复合型高层次女性人才，提高妇女参政议政的意识和竞争能力。加强对女性专业技术人员和管理人员的培训，提高其政治素质和业务能力。
　　--重视奖励、表彰妇女中的先进典型人物，大力宣传妇女在两个文明建设与社会发展中的作用和贡献，提高女性人才在社会上的影响力。

　　**三、**妇女与教育
　　（一）主要目标。
　　1.保障女童接受九年义务教育的权利。小学适龄女童的净入学率达到99%左右，小学5年巩固率提高到95%左右，基本杜绝小学适龄女童失学。初中女童毛入学率达到95%左右。
　　2.高中阶段教育女性毛入学率达到75%左右，高等教育女性毛入学率达到15%左右。
　　3.成人妇女识字率提高到85%以上，其中青壮年妇女识字率提高到95%左右。
　　4.提高妇女的终身教育水平。
　　5.妇女平均受教育年限达到发展中国家的先进水平。
　　（二）策略措施。
　　缩小男女受教育差距、提高妇女的科学文化素质是妇女发展的决定性因素。
　　1.国家宏观政策。
　　国家的人才发展战略要体现男女平等原则，将妇女教育的主要目标纳入国家的教育发展规划。
　　--在课程、教育内容和教学方法改革中，把社会性别意识纳入教师培训课程，在高等教育相关专业中开设妇女学、马克思主义妇女观、社会性别与发展等课程，增强教育者和被教育者的社会性别意识。
　　--制定相关政策，提供妇女享有与男子平等的受教育的机会和途径，缩小男女受教育差距。
　　--改善学科领域的性别分布结构，培养高新技术和现代管理领域的女性专业人才。
　　--在实现教育技术现代化和教育信息化的过程中，保障妇女与男子共享信息和优质教育资源。
　　--加大对贫困地区教育的投入，为贫困地区妇女受教育创造条件。
　　2.法律和部门政策。
　　制定和完善有利于妇女与男子接受同等教育的相关法律法规和政策。
　　--教育立法要体现性别平等，保障妇女受教育的权利。
　　--进一步贯彻落实《[中华人民共和国义务教育法](https://www.pkulaw.com/chl/27bc0f58a13f5a17bdfb.html)》等相关法律法规，重点解决西部贫困地区和少数民族地区女童、残疾女童、流动人口中女童的义务教育问题。帮助失、辍学女童完成九年义务教育。缩小男女童受教育差距。
　　--发挥大中城市和经济发达地区的优势，提高内地学校培养少数民族女学生的比例。
　　--提高妇女接受职业教育和成人教育的水平，注重培养妇女的职业技能和适应职业变化的能力。通过正规的学历教育与非学历职业教育以及各种培训，使新增女性劳动力和在职女职工能够普遍接受各种形式的职业教育和成人教育。重点发展县（市）、乡（镇）和农村的中等职业教育，为初中毕业生中的女性提供多种形式的继续学习机会。
　　--扩大妇女接受高等教育的规模。全面落实各项资助经济困难学生的政策，帮助贫困女大学生完成学业。
　　--积极利用网络和现代远程教育资源，为妇女接受教育创造条件和机会。
　　--加大扫除妇女文盲工作的力度，把扫除农村妇女文盲作为扫盲工作的重点。
　　--把妇女的素质教育贯穿于正规教育和非正规教育以及培训等各个方面，培养女学生的知识创新能力、社会适应能力、人文素养和科学精神，普遍提高妇女的文化知识水平和科学技术的应用能力。
　　--提高妇女终身教育水平。逐步形成大众化、社会化的终身教育体系，为妇女提供终身学习的条件和机会。
　　--努力创造条件，使农村妇女劳动者能普遍受到实用生产技术培训和文化知识教育。在初中教育阶段，将基础教育与“绿色证书”教育有机结合，使女学生获得更全面的知识和劳动技能。
　　--通过对口扶贫支教、启动远程教育扶贫项目等措施，提高边远贫困地区妇女受教育的水平。
　　--对残疾妇女进行职业教育、职业培训，为残疾妇女提供受教育的机会，提高其受教育程度，增强其生存和发展能力。
　　3.社会教育和培训。
　　广泛宣传性别平等和有关教育的法律法规，创造有利于妇女接受教育的社会环境。
　　--鼓励和支持社会力量办学，为妇女接受教育创造条件和机会。
　　--继续发动社会力量参与妇女扫盲。

　　**四、**妇女与健康
　　（一）主要目标。
　　1.妇女在整个生命周期享有卫生保健服务，提高妇女的预期寿命。
　　2.提高妇女生殖健康水平。
　　3.保障妇女享有计划生育的权利。
　　4.流动人口中的妇女享有与户籍所在地妇女同等的卫生保健服务。
　　5.将妇女艾滋病病毒感染率控制在较低水平。
　　6.提高妇女的健身意识，增强妇女身体素质。
　　（二）策略措施。
　　妇女身体、精神和社会适应能力的完全健康状态是反映妇女生存状况的基本指标。
　　1.国家宏观政策。
　　在国家的卫生改革与发展规划中体现妇女健康的主要目标。
　　--妇女卫生保健以预防为主，以农村为重点。
　　--在优化卫生资源配置中，合理安排妇女卫生保健服务经费和科研经费。
　　--引导卫生保健产业发展，不断满足妇女的健康需求。
　　--通过宣传教育，在全社会树立正确的妇女健康观念，普及健康知识。
　　--完善计划生育管理和技术服务体系，提倡避孕节育的知情选择，强化夫妻共同承担计划生育的责任。
　　2.法律和部门政策。
　　贯彻落实《[中华人民共和国母婴保健法](https://www.pkulaw.com/chl/ea633acd04298a40bdfb.html)》，不断完善妇幼卫生法律法规及政策，保护妇女的健康权利。
　　--加快卫生立法，完善和落实以公共卫生和妇女健康为主要内容的法律法规。
　　--强化卫生行政执法，加大对卫生保健产品、卫生机构和专业人员的监督管理，依法查处各类危害妇女身体健康的违法行为。
　　--强化社区在健康服务中的作用，把人口与计划生育工作纳入社区管理和服务体系，建立以现居住地为主的管理模式。将流动人口孕产妇保健纳入流入地孕产妇保健范围。
　　--加强农村卫生组织建设，完善卫生服务网，做好预防保健的综合服务，重点筛查和治疗严重危害农村妇女健康的疾病，预防和减少农村妇女妇科病的发生。
　　--对妇幼卫生人员进行卫生保健专业知识培训，强化基本知识和基本技能，提高卫生保健技术水平和服务质量。
　　--针对妇女不同时期生理和心理特点提供生殖保健服务和精神健康服务，提高妇科常见病的普查普治率。
　　--加强产科建设，创造住院分娩条件，使全国孕产妇死亡率以2000年为基数下降1/4。农村孕产妇住院分娩率达到65%，高危孕产妇住院分娩率达到90%以上。住院分娩确有困难的边远地区，消毒接生率达到95%以上。
　　--提高产科质量，减少不必要的医学干预，降低剖宫产率。
　　--开展生殖保健科学研究，提高对严重危害妇女健康疾病的预防和治疗水平。
　　--研究推广安全、有效的避孕节育新技术、新方法，提供避孕节育的优质服务。节育手术并发症发生率控制在1‰以下。预防意外妊娠，降低人工流产率。
　　--开发、研制男性避孕节育产品，动员男性采取节育措施，提高男性避孕方法使用比重。
　　--以生殖健康教育为中心，普及生殖保健、优生优育、避孕节育知识，生殖保健知识普及率和育龄人口计划生育知识普及率达到80%以上。
　　--加强艾滋病防治工作，强化对采供血机构和血制品生产单位的管理；利用宣传教育网络，全民普及艾滋病防治及自我防范知识，提高艾滋病知识知晓率。
　　3.社会保障和服务。
　　完善医疗保障制度，保障妇女享有基本的医疗服务。
　　--促进城镇职工基本医疗保险、生育保险制度的进一步完善。
　　--通过合作医疗等多种形式的健康保障制度，提高农村妇女享受卫生保健的水平和抵御疾病风险的能力。
　　--实现医疗卫生服务观念和服务模式从以病为本向以人为本的根本转变，治病与保健相结合，不断满足广大妇女的健康需求。
　　--倡导科学文明的健身方式，提高妇女身体素质。利用现有体育设施以及在社区、公园兴建和开辟健身场所，为妇女参与全民健身运动创造条件。

　　**五、**妇女与法律
　　（一）主要目标。
　　1.健全和完善促进男女平等的法律法规。
　　2.开展维护妇女权益法律法规的宣传教育。
　　3.保护妇女的人身权利，禁止针对妇女的一切形式的暴力。
　　4.维护妇女与男子平等的财产权利。
　　5.保护妇女合法的控告、申诉权及在诉讼中的各项权益。
　　6.为妇女提供法律援助。
　　（二）策略措施。
　　完善立法并保障法律法规赋予妇女的各项权利是实现妇女合法权益的重要保障。
　　1.立法。
　　在国家立法中充分体现社会性别意识，规范影响妇女发展的社会行为。
　　--以《[中华人民共和国宪法](https://www.pkulaw.com/chl/a3b96c9f520bcc3bbdfb.html)》和《[中华人民共和国妇女权益保障法](https://www.pkulaw.com/chl/dff2eefbddf155b3bdfb.html)》为依据，进一步加强和完善保护妇女合法权益的专门立法。
　　--在健全和完善相关法律法规的过程中关注妇女问题，保障妇女在政治、经济、文化、社会和家庭生活等方面享有与男子平等的权利。
　　2.司法和执法。
　　强化司法保护和法律监督，加大执法力度。
　　--有效预防、严厉打击各种侵害妇女人身权利和财产权利的违法犯罪行为，降低强奸、拐卖等侵害妇女人身权利的刑事案件的发案率，提高结案率。预防和制止针对妇女的家庭暴力。严厉打击嫖娼卖淫活动，扫除黄、赌、毒等社会丑恶现象。
　　--建立和完善妇联特邀陪审员制度，切实贯彻男女平等、保护妇女合法权益的原则，保障涉及妇女的案件在审判过程中的公正。
　　--加强法律监督，维护司法公正，提高司法效率。
　　3.法律知识的宣传教育。
　　把宣传保护妇女权益的法律法规纳入国家法制宣传教育规划。
　　--把普法宣传和教育纳入精神文明建设规划，提高全社会维护妇女合法权益的法律意识和法律素质，提高维护妇女权益法律法规知识的普及率。
　　--提高妇女的法律意识，增强妇女维护自身权利的能力。
　　4.法律服务和援助。
　　建立法律援助体系，为受害妇女提供多种形式的法律服务和社会救助。
　　--健全信访接待制度，开展法律咨询和服务，及时处理受害妇女的投诉。
　　--采取多种形式，向遭受暴力侵害和需要帮助的妇女提供法律、医疗、精神康复等方面的帮助和服务。
　　--妇联、工会、共青团在各自的职责范围内依法维护受害妇女的合法权益。

　　**六、**妇女与环境
　　（一）主要目标。
　　1.创造有利于妇女全面发展的社会环境。
　　2.提高妇女享有社会福利的水平。
　　3.加强家庭美德建设，大力倡导建立平等、文明、和睦、稳定的家庭关系。
　　4.为妇女创造适宜的生活和工作环境。
　　5.提高妇女参与环境保护及决策的程度。
　　6.增加妇女自我支配的时间。
　　（二）策略措施。
　　优化社会环境、保护自然环境是有效保障妇女合法权益、促进妇女进步与发展的要素。
　　1.国家宏观政策。
　　在文化、教育、宣传、环境等发展规划中充分体现妇女与环境的主要目标。
　　--制定具有社会性别意识的文化和传媒政策，加大男女平等基本国策的宣传力度，增强全社会的社会性别意识，逐步消除对妇女的偏见、歧视以及贬抑妇女的社会观念，为妇女发展创造良好的社会环境。
　　--鼓励妇女成为有理想、有道德、有文化、有纪律和自尊、自信、自立、自强的新女性。
　　--加大对社会福利事业的投入，加快社会福利机构的建设和设施改造，完善低收入妇女群体的生活保障制度，逐步提高城市妇女贫困人口的救济补助标准。
　　--积极发展老龄事业和产业，逐步实现老年服务社会化，保障老年妇女的身心健康。
　　--发挥妇女在环境保护特别是农村环境保护方面的重要作用，提高妇女参与环境保护及决策的程度。
　　--扩大妇女工作领域的国际交流与合作，提高我国妇女在国际事务中的影响力，促进世界和平、进步与发展。
　　2.法律与部门政策。
　　--在新闻出版、广播影视以及文学艺术等领域展现妇女在经济发展和社会进步中的成就和作用，大力宣传妇女中的先进模范人物。
　　--加强文化市场管理，禁止在宣传媒体、广告和文艺作品中出现色情或有辱妇女人格的作品。
　　--为妇女在新闻宣传领域的发展提供更多的条件和机会，使妇女广泛参与宣传媒体的管理、制作、教育、培训和研究。提高妇女对宣传媒体资源的占有程度。
　　--加强家庭美德建设，倡导建立民主、平等、尊重妇女、尊老爱幼的家庭关系，倡导科学文明健康的生活方式，鼓励夫妻共同承担家务劳动。
　　--强化城市环境综合治理，减少家庭、工作场所以及其他环境中有毒有害物质对妇女造成的危害，提高垃圾无害化处理率和污水集中处理率。
　　--把农村自来水建设和卫生厕所建设纳入小城镇建设规划。提高农村缺水地区供水受益率和农村改水受益率、自来水普及率。加强农村改厕技术指导，提高农村卫生厕所普及率。
　　--提高妇女的环境保护意识和环境保护基本知识的普及率。引导妇女积极参加生态环境保护，改变对环境有害的生产和生活方式，保护饮用水源，防治农业化学污染，发展生态农业和绿色产业。倡导绿色消费。
　　3.社会保障和服务。
　　--在城镇建立以基本养老保险、基本医疗保险和有关商业保险、社会救助、社会福利为主要内容的保障体系；在农村以家庭养老为主，逐步扩大社会养老保障的覆盖面。
　　--加强老年文化体育活动场所建设，丰富老年妇女的精神文化生活。
　　--在社区优先发展对家庭生活有直接影响的公共服务，实现家务劳动社会化，逐步增加妇女的自我支配时间。

　　组织与实施

　　**一、**国务院妇女儿童工作委员会负责[《纲要》](https://www.pkulaw.com/chl/37733dd7246afab4bdfb.html)的组织实施。国务院各有关部门和社会团体要根据[《纲要》](https://www.pkulaw.com/chl/37733dd7246afab4bdfb.html)的要求并结合各自的职责，制定相应的实施方案。

　　**二、**地方各级政府要结合实际制定本地区妇女发展规划，并纳入当地经济和社会发展的总体规划，统一部署，统筹安排。要将[《纲要》](https://www.pkulaw.com/chl/37733dd7246afab4bdfb.html)的实施纳入政府的议事日程，纳入政府主要负责人和主管负责人的政绩考核。

　　**三、**建立健全实施[《纲要》](https://www.pkulaw.com/chl/37733dd7246afab4bdfb.html)的工作机制。国务院各有关部门和社会团体每年要向国务院妇女儿童工作委员会报告实施[《纲要》](https://www.pkulaw.com/chl/37733dd7246afab4bdfb.html)的工作情况。地方各级妇女儿童工作委员会要建立相应的工作制度和报告制度。要加强调查研究，坚持分类指导、示范先行的工作方法，及时掌握有关情况，总结推广经验。要注重对妇女领域的理论研究。

　　**四、**各级政府应根据财力情况，合理安排实施[《纲要》](https://www.pkulaw.com/chl/37733dd7246afab4bdfb.html)所需经费。要多渠道筹集资金，重点扶持贫困地区和少数民族地区妇女事业的发展。

　　监测与评估

　　**一、**对[《纲要》](https://www.pkulaw.com/chl/37733dd7246afab4bdfb.html)的实施情况实行分级监测评估。要加强妇女发展综合统计工作，增设分性别统计指标，建立和完善分性别数据库。做好信息收集、整理、反馈和交流工作，分析妇女发展现状，预测趋势，评估实施效果，为制定规划、科学决策提供依据。

　　**二、**建立国家和省（自治区、直辖市）的妇女状况监测体系，制定切实可行、科学规范的监测评估方案，全面、动态地监测妇女发展状况。[《纲要》](https://www.pkulaw.com/chl/37733dd7246afab4bdfb.html)分性别的统计指标要纳入国家统计制度和各有关部门的常规统计和统计调查。建立健全劳动监察、卫生监测、教育督导、统计评估、法律监督机构，完善监测机制，确保[《纲要》](https://www.pkulaw.com/chl/37733dd7246afab4bdfb.html)的有效实施。

　　**三、**建立定期报送和审评制度。国务院妇女儿童工作委员会各成员单位和各有关部门，每年要向国务院妇女儿童工作委员会办公室和国家统计局报送监测数据及目标实施进展情况，并对报送的监测数据进行分析，评估实施效果。国家监测评估周期分为年度监测、每3-5年的阶段性监测评估和10年的终期监测评估。

　　**四、**建立监测评估机构。国务院妇女儿童工作委员会设立监测评估领导小组，负责审批监测评估方案，根据监测评估结果提出相应对策。
　　监测评估领导小组下设统计监测组和专家评估组。
　　统计监测组由国家统计局牵头，相关部门共同组成。负责制定[《纲要》](https://www.pkulaw.com/chl/37733dd7246afab4bdfb.html)分性别的统计监测指标体系，提出监测的重点指标；确定监测方法，收集监测数据，建立和完善分性别数据库；向国务院妇女儿童工作委员会提交全国的妇女状况统计监测报告；指导各地区做好实施[《纲要》](https://www.pkulaw.com/chl/37733dd7246afab4bdfb.html)的统计监测工作。
　　专家评估组由国务院妇女儿童工作委员会办公室牵头，相关部门推荐专家组成。负责制定检查评估方案；审评监测报告，并对重点、难点问题的解决提出意见和建议；开展阶段性评估，向国务院妇女儿童工作委员会提交评估报告；指导各地区对实施[《纲要》](https://www.pkulaw.com/chl/37733dd7246afab4bdfb.html)的检查评估工作。
　　各地区也要建立相应的监测评估机构和制度，及时、准确、全面地反映[《纲要》](https://www.pkulaw.com/chl/37733dd7246afab4bdfb.html)和本地区规划的实施情况。

中国儿童发展纲要
（2001-2010年）

　　前言

　　当今世界，新科技革命迅猛发展，经济全球化趋势增强，综合国力竞争日趋激烈。2001-2010年是我国经济和社会发展极为重要的时期。推进社会主义现代化建设，实现经济和社会的全面进步，必须把提高国民素质、开发人力资源作为战略任务；必须从儿童早期着手，培养、造就适应新世纪需要的高素质人才队伍。
　　儿童期是人的生理、心理发展的关键时期。为儿童成长提供必要的条件，给予儿童必需的保护、照顾和良好的教育，将为儿童一生的发展奠定重要基础。
　　1992年，我国参照世界儿童问题首脑会议提出的全球目标和《儿童权利公约》，从中国国情出发，发布了《九十年代中国儿童发展规划纲要》。这是我国第一部以儿童为主体、促进儿童发展的国家行动计划。各级政府和有关部门坚持“儿童优先”的原则，加强领导，强化责任，制定政策，采取措施，认真实施，基本实现了《九十年代中国儿童发展规划纲要》提出的主要目标，使我国儿童生存、保护和发展取得历史性的进步。
　　--婴儿死亡率、5岁以下儿童死亡率分别从90年代初的51‰和61‰下降到2000年的32.2‰和39.7‰。
　　--孕产妇死亡率从1989年的94.7/10万下降到2000年的53.0/10万。
　　--5岁以下儿童低体重患病率从1990年的21%下降到10%。
　　--基本普及了食盐加碘。
　　--儿童计划免疫接种率以县为单位达到90%以上；实现了无脊髓灰质炎的目标。
　　--农村改水受益人口覆盖率和卫生厕所普及率2000年分别达到92.38%和44.84%。
　　--小学适龄儿童净入学率由1990年的96.3%提高到2000年的99.1%，男女童入学差异由1990年的2.91个百分点下降到2000年的0.07个百分点；小学5年巩固率由1990年的71.4%提高到2000年的94.5%，占全国人口85%的地区普及了九年义务教育。
　　--青壮年文盲率2000年下降到5%以下。
　　但是，作为人口众多的发展中国家，我们仍然面临诸多的问题和挑战，如：儿童发展的整体水平仍然需要提高，儿童发展的环境需要进一步优化；地区之间、城乡之间儿童生存、保护和发展的条件、水平存在明显差异；贫困尚未消除，仍有数百万儿童生活在贫困中；随着流动人口数量的增加、城镇化水平的提高和农村人口的转移，这些人群中儿童的保健、教育、保护问题亟待解决；艾滋病病毒携带者和艾滋病患者中的儿童数量呈上升趋势；侵害儿童权益的违法犯罪行为时有发生。因此，改善儿童生存、保护和发展条件，促进儿童健康成长，仍然是今后一个时期的重要任务。
　　《中国儿童发展纲要（2001-2010年）》（以下简称[《纲要》](https://www.pkulaw.com/chl/37733dd7246afab4bdfb.html)）按照《[中华人民共和国国民经济和社会发展第十个五年计划纲要](https://www.pkulaw.com/chl/f439200c548488c1bdfb.html)》的总体要求，根据我国儿童发展的实际情况，以促进儿童发展为主题，以提高儿童身心素质为重点，以培养和造就21世纪社会主义现代化建设人才为目标，从儿童与健康、儿童与教育、儿童与法律保护、儿童与环境4个领域，提出了2001-2010年的目标和策略措施。
　　[《纲要》](https://www.pkulaw.com/chl/37733dd7246afab4bdfb.html)的发布和实施，必将进一步促进我国儿童的健康成长和儿童事业的持续发展。

　　总目标

　　坚持“儿童优先”原则，保障儿童生存、发展、受保护和参与的权利，提高儿童整体素质，促进儿童身心健康发展。儿童健康的主要指标达到发展中国家的先进水平；儿童教育在基本普及九年义务教育的基础上，大中城市和经济发达地区有步骤地普及高中阶段教育；逐步完善保护儿童的法律法规体系，依法保障儿童权益；优化儿童成长环境，使困境儿童受到特殊保护。

　　主要目标与策略措施

　　**一、**儿童与健康
　　改善儿童卫生保健服务，提高儿童健康水平。
　　（一）主要目标。
　　1.提高出生人口素质。
　　--婚前医学检查率城市达到80%，农村达到50%。
　　--减少出生缺陷的发生。
　　2.保障孕产妇安全分娩。
　　--孕产妇死亡率以2000年为基数下降1/4。
　　--农村孕产妇住院分娩率达到65%，高危孕产妇住院分娩率达到90%以上，农村消毒接生率达到95%以上。
　　--孕产妇缺铁性贫血患病率以2000年为基数下降1/3。
　　--孕产妇保健覆盖率在城市达到90%以上，在农村达到60%以上。
　　3.降低婴儿和5岁以下儿童死亡率。
　　--婴儿和5岁以下儿童死亡率以2000年为基数分别下降1/5。
　　--降低新生儿窒息和5岁以下儿童肺炎、腹泻等构成主要死因的死亡率。
　　--新生儿破伤风发病率以县为单位降低到1‰以下。
　　--免疫接种率以乡（镇）为单位达到90%以上。将乙肝疫苗接种纳入计划免疫，并逐步将新的疫苗接种纳入计划免疫管理。
　　4.提高儿童营养水平，增强儿童体质。
　　--5岁以下儿童中、重度营养不良患病率以2000年为基数下降1/4。
　　--低出生体重发生率控制在5%以下。
　　--婴幼儿家长的科学喂养知识普及率达到85%以上。
　　--婴儿母乳喂养率以省（自治区、直辖市）为单位达到85%，适时、合理添加辅食。
　　--减少儿童维生素A缺乏。
　　--合格碘盐食用率达到90%以上。
　　--儿童保健覆盖率在城市达到90%以上，在农村达到60%以上，逐步提高女童及流动人口中儿童保健覆盖率。
　　--中小学生《[国家体育锻炼标准](https://www.pkulaw.com/chl/6caa4f0165ffff5dbdfb.html)》及格率达到90%以上。
　　5.加强儿童卫生保健教育。
　　--减少未成年人吸烟，预防未成年人吸毒。
　　--预防和控制性病、艾滋病、结核病的蔓延和增长。
　　--提供多种形式的儿童心理健康咨询及不良心理矫正服务。
　　（二）策略措施。
　　1.国家宏观政策。
　　努力创造条件，让儿童享有可达到的最高标准的健康服务。
　　--深化医疗卫生改革，努力实现儿童健康的主要目标。
　　--合理安排和增加妇幼卫生、疾病预防控制等基本卫生服务经费投入。
　　--加强妇幼卫生保健知识的教育、培训和宣传。
　　2.法律和部门政策。
　　完善和落实关于妇幼卫生保健的法律法规和政策措施。
　　--认真贯彻《[中华人民共和国母婴保健法](https://www.pkulaw.com/chl/ea633acd04298a40bdfb.html)》等法律法规，加强执法监督。
　　--发展社区卫生服务，加强农村卫生服务网的建设和规范化服务，提供孕产妇和儿童保健、儿童生长发育监测、计划免疫、儿童常见病诊疗等基本卫生服务。
　　--全面开展妇女生殖保健服务，加强对孕产妇产前、产时和产后的保健服务。在城乡特别是农村继续实施“母亲安全”项目，开展高危孕产妇的筛查，提高孕产妇住院分娩率，创造安全分娩的必要条件，降低孕产妇死亡率。
　　--积极防治儿童多发病和常见病，重视做好儿童眼、口腔和听力保健等工作。
　　--加强冷链系统建设，增强常规免疫的有效性，逐步增加接种疫苗种类。预防性注射应达到安全注射标准。
　　--倡导科学喂养和良好的饮食习惯，改善儿童营养。支持母乳喂养。在贫困地区和维生素A缺乏地区，做好儿童维生素A缺乏的干预工作。对中小学生提倡饮用畜奶，分步实施国家“学生饮用奶计划”，推行“国家大豆行动计划”。有计划、有步骤地普及学生营养餐，减少营养不良或营养过剩。
　　--加强儿童卫生保健的理论研究与技术推广。继续推广和应用计划免疫、口服补液疗法、儿童疾病综合管理、婴幼儿科学喂养、食盐加碘、儿童生长发育监测等适宜技术。研究减少新生儿疾病、儿童营养不良、儿童意外伤害等预防措施。
　　--广泛开展宣传教育和科学普及工作，提高妇女儿童自我保健和利用卫生服务的能力。倡导科学、文明、健康的生活方式。开展有关性病和艾滋病的危害、预防及自我防范知识的宣传工作；进一步重视在青少年中进行青春期教育、预防吸烟和吸毒的教育。
　　3.社会保障和服务。
　　进一步完善医疗保障制度，确保儿童享有基本卫生医疗和保健服务。
　　--健全农村初级卫生保健服务体系，因地制宜发展合作医疗以及多种形式的健康保障制度，提高儿童保健水平和抵御疾病风险的能力。
　　--多渠道设立贫困家庭的疾病救助基金，帮助特困家庭孕产妇和儿童获得必要的医疗救助。加强对弃婴和孤儿的医疗救助。对城市流动人口中的孕产妇、儿童逐步实行保健管理。
　　--重视儿童体育。有条件的城市社区、乡（镇）要为儿童健身提供必要的体育设施。培养儿童良好的体育锻炼习惯，学校保证中小学生每天1小时的体育锻炼时间。
　　--重视儿童心理卫生知识的普及。在学校开设心理健康课程；逐步在大中城市和其他有条件的地方建立儿童心理咨询和矫正服务机构。

　　**二、**儿童与教育
　　保障儿童受教育权利，提高儿童受教育水平。
　　（一）主要目标。
　　1.全面普及九年义务教育，保障所有儿童受教育的权利。
　　--小学适龄儿童净入学率达到99%左右，小学5年巩固率提高到95%左右。
　　--初中毛入学率达到95%左右。
　　--发展特殊教育。
　　--流动人口中的儿童基本能接受九年义务教育。
　　2.适龄儿童基本能接受学前教育。
　　--发展0-3岁儿童早期教育。
　　--大中城市和经济发达地区适龄儿童基本能接受学前3年教育，农村儿童学前1年受教育率有较大提高。
　　3.有步骤地普及高中阶段教育。
　　--全国高中阶段毛入学率达到80%以上，大中城市和经济发达地区普及高中阶段教育。
　　4.提高教育质量和效益。
　　--建立适应21世纪需要的现代化基础教育课程体系。
　　--改革考试评价制度。
　　5.提高家庭教育水平。
　　--建立多元化的家长学校办学体制，增加各类家长学校的数量。
　　--提高儿童家长家庭教育知识的知晓率。
　　（二）策略措施。
　　1.国家宏观政策。
　　落实教育优先发展战略，把提高儿童整体素质作为人才战略的基础工程。
　　--保证教育与国民经济和社会发展相适应，并适度超前发展。
　　--合理配置教育资源，缩小地区差距，为所有儿童提供平等的受教育机会和条件，确保教育特别是义务教育的公平、公正。
　　--完善教育投入机制，增加教育投入。
　　2.法律和部门政策。
　　完善并落实教育法律法规和政策。
　　--进一步加快教育立法工作，完善教育法律体系。
　　--加大教育执法力度，健全教育督导机构和制度，提高全社会的教育法制观念和有关法律知识水平，积极推进依法治教。
　　--全面贯彻国家教育方针，整体推进素质教育，促进学生德、智、体、美全面发展。注重培养学生的创新意识、实践能力和科学精神。
　　--继续减轻中小学生的课业负担，组织学生参加社会实践活动和社会公益活动，培养社会实践能力。将中小学综合实践基地建设纳入地方发展规划。
　　--将性别平等意识纳入教育内容。
　　--实施“国家贫困地区义务教育工程”、“西部教育开发工程”、“希望工程”和“春蕾计划”等助学工程，保障贫困地区、少数民族地区儿童就学权利。
　　--切实保障女童受教育的权利，消除阻碍女童入学的障碍。
　　--切实保障残疾儿童、孤儿和流动人口中儿童受教育的权利。使残疾儿童与其他儿童同步接受义务教育；贯彻落实孤儿就学的有关优惠政策；完善流动人口中儿童就学制度；根据国家推进城镇化的要求，做好教育规划，满足农村适龄儿童向城镇转移后的就学需要。
　　--发展学前教育。建立并完善0-3岁儿童教育管理体制。合理规划并办好教育部门举办的示范性幼儿园，同时鼓励社会多渠道、多形式发展幼儿教育。积极探索非正规教育形式，满足边远、贫困地区及少数民族地区幼儿接受学前教育的需要。
　　--实施“教育信息化工程”，推动中小学校普及计算机等信息技术教育。建设现代远程教育网络，发挥卫星电视教育的作用，重点满足边远山区、海岛等地区的教育需要。
　　--提高教师队伍整体素质。提升教师学历合格率和学历层次，重视中小学校长和教师的在职培训和继续教育。加强师德建设。
　　--学校、托幼园所的教职工爱护、尊重儿童，维护儿童的人格尊严，不得歧视、体罚或变相体罚儿童。中、小学校不得随意开除学生。学校纪律、教育方法应适合学生身心特点。
　　--发挥学校、家庭、社会各自的教育优势，充分利用社会资源形成教育合力，促进学校教育、家庭教育、社会教育的一体化。
　　--重视和改进家庭教育。加强家庭教育知识的宣传和理论研究。办好各类家长学校，帮助家长树立正确的保育、教育观念，掌握科学的教育知识与方法。

　　**三、**儿童与法律保护
　　完善和落实有关法律法规，依法保障儿童权益。
　　（一）主要目标。
　　1.依法保障儿童生存权、发展权、受保护权和参与权。
　　2.依法打击侵害儿童合法权益的违法犯罪行为。
　　--控制并减少侵害儿童人身权利的各类刑事案件。
　　--禁止虐待、溺弃儿童，特别是女婴和病残儿童。
　　--禁止使用童工（未满16周岁）和对儿童的经济剥削。
　　3.预防和控制未成年人犯罪。
　　--控制未成年人犯罪率并减少重新犯罪率。
　　--中小学校普遍进行法律知识教育。
　　4.在诉讼中依法维护未成年人的合法权益。
　　--保障未成年人参加诉讼和辩护的权利。
　　--基层法院建立少年法庭，对未成年人犯罪案件和涉及未成年人犯罪案件不公开审理，或采取适当的回避制度。
　　5.建立法律援助机构，为儿童提供法律援助。
　　（二）策略措施。
　　1.立法与执法。
　　完善有关儿童的立法，强化执法，有效保障儿童权益。
　　--完善儿童权益保护的法律法规，建立健全执法监督机制。认真执行《[中华人民共和国未成年人保护法](https://www.pkulaw.com/chl/f676777bde217880bdfb.html)》、《[中华人民共和国预防未成年人犯罪法](https://www.pkulaw.com/chl/1137d4a7728b3300bdfb.html)》、《[中华人民共和国收养法](https://www.pkulaw.com/chl/5b741c5640fd501dbdfb.html)》等法律法规。认真履行《儿童权利公约》。
　　--严厉打击杀害、强奸、摧残、虐待、拐卖、绑架、遗弃等侵害儿童人身权利和引诱、教唆或强迫未成年人犯罪的刑事犯罪。保护儿童免遭一切形式的性侵犯。严禁利用儿童生产和贩运毒品。
　　--加强对企业用工的管理和监督，及时发现和查处使用童工现象。
　　2.司法保护。
　　在处理未成年人违法犯罪时，落实司法保护原则。
　　--对违法犯罪的未成年人实行教育、感化、挽救的方针，坚持教育为主，惩罚为辅的原则。
　　--公安机关和法院、检察院在办理未成年人刑事案件中要相互配合，在侦查、起诉、审判各诉讼阶段依法给予未成年犯罪嫌疑人或被告人特殊的、有别于成年人的待遇。对未成年人罪犯执行刑罚时，要与成年人罪犯分别关押、管理和教育。
　　--对检察院不起诉、法院免于刑事处罚或者宣告缓刑，以及被解除收容教养或者服刑期满释放的未成年人复学、升学、就业不得歧视。
　　3.法律宣传与服务。
　　通过宣传教育和为儿童提供法律服务，动员全社会重视和保护儿童权益。
　　--宣传普及保护儿童的法律知识，提高全社会保护儿童合法权益的责任意识。有关部门、学校、家庭应密切配合，共同做好儿童保护工作。
　　--加强对执法和司法人员有关儿童法律知识、权益保护和办案技能的培训，提高执法水平。
　　--教育儿童父母或其他监护人依法履行监护职责和抚养义务。禁止对儿童实施家庭暴力及其他形式的身心虐待。禁止强迫未成年人结婚或为未成年人订立婚约。
　　--加强对儿童的纪律教育、法制教育和安全教育，增强儿童法律意识，提高儿童自我保护和防范能力。
　　--各类媒体在报道有关未成年人案件时不得公开其真实姓名和身份。
　　--建立保护儿童权益的工作网络。防止侵害儿童合法权益的行为发生；及时矫治未成年人的不良行为，预防和减少未成年人违法犯罪行为；做好违法犯罪未成年人的教育挽救工作和遭受侵害未成年人的身心康复、回归社会工作。
　　--积极设立面向儿童的法律援助机构，完善法律援助体系和网络，为儿童提供多种形式的法律援助服务。

　　**四、**儿童与环境
　　改善儿童生存和发展环境，尊重并鼓励儿童积极参与。
　　（一）主要目标。
　　1.改善儿童生存的自然环境。
　　--提高农村缺水地区供水受益率和农村改水受益率、自来水普及率。
　　--提高农村卫生厕所普及率。
　　--城市空气和水按功能区基本达到国家环境保护标准，提高城市垃圾无害化处理率和污水集中处理率。
　　--增加森林和绿地面积。
　　2.优化儿童发展的社会环境。
　　--尊重、爱护儿童，使儿童免受一切形式的歧视和伤害。
　　--为儿童提供必需的闲暇、娱乐时间，保障儿童参与家庭、社会和文化生活的权利。
　　--提高儿童食品、玩具、用具和游乐设施的质量，保证其安全无害。
　　--为儿童提供健康向上的精神产品，净化儿童成长的文化环境。
　　--各类媒体传播有益于儿童健康成长的社会、文化信息，保护儿童免受不良信息影响。
　　--为儿童成长创造良好的家庭环境。
　　--增加社区儿童教育、科技、文化、体育、娱乐等课外活动设施和场所，90%以上的县（市）至少有1处儿童校外活动场所。
　　3.保护处于困境中的儿童。
　　--提高残疾儿童康复率。
　　--改善孤儿、弃婴的供养、教育、医疗康复状况。
　　--基本达到每个地级市有1所具有养护、医疗康复、教育能力的儿童福利院。
　　（二）策略措施。
　　1.国家宏观政策。
　　国家制定相关法律法规和政策时要体现“儿童优先”原则，有利于儿童发展。
　　--在全社会树立尊重儿童、爱护儿童、教育儿童的良好风尚，保障儿童参与的权利。
　　--将儿童发展纳入国民经济和社会发展总体规划，加大对儿童事业的投入。
　　--鼓励儿童积极参与家庭、文化和社会生活，培养儿童做有理想、有道德、有文化、有纪律的“四有”新人。
　　--建立和完善儿童社会保障制度。
　　--关注女童和处于特殊困境的儿童，保证其获得健康成长和平等发展的机会。
　　--扩大儿童工作领域的国际交流与合作，积极参与全球和区域性的国际交流和合作。
　　2.法律和部门政策。
　　完善和落实有利于儿童生存、保护和发展的相关法律法规和政策。
　　--依法加强生态建设，加大环境保护和治理的力度。植树造林，治理大气、水、固体废物和噪音等环境污染，控制和治理工业、生活、农村面源污染，保护饮用水源。在农村继续以改水、改厕为重点，带动环境卫生整治，加强卫生厕所建设和人畜粪便无害化处理等技术指导和服务，推广圈养家禽家畜。为儿童的健康成长创造良好的自然环境。
　　--加强儿童食品、用品、玩具和游乐设施生产、销售的监督管理，完善检测标准和质量认证工作。加强公共设施的管理和安全监察，宣传安全知识，提高儿童及家长的安全意识，减少儿童意外伤害。鼓励企业在经营活动中坚持儿童受惠原则，为儿童发展提供支持。
　　--各类媒体应为儿童健康成长创造良好的舆论环境。鼓励创作优秀儿童图书、影视、歌曲、舞蹈、戏剧、美术等作品，宣传积极向上的儿童形象，丰富儿童精神生活。对文化市场进行规范化管理，打击非法出版物，禁止各类媒体传播色情、暴力等损害儿童身心健康的信息，减少对儿童的负面影响。加强对学校周边经营场所的监督检查。
　　--积极开展文明家庭创建活动，加强家庭美德建设，倡导平等、文明、和睦、稳定的家庭关系，为儿童健康成长营造良好的家庭环境。
　　--增加儿童课外活动设施和场所，将儿童校外教育、科技、文化、体育、娱乐等设施建设纳入城镇建设规划。多渠道筹集资金，重点资助中、西部地区的青少年活动场所建设。强化社区对儿童的服务、管理和教育功能。加强农村儿童科技、文化园建设。加强对儿童活动场所的管理，提高社会效益，逐步实现社会公益活动场所面向儿童免费开放。
　　3.社会保障与服务。
　　完善社会保障机制，促进困境儿童的生存与发展。
　　--加大儿童福利事业的投入，改善设施，为孤儿、残疾儿童、弃婴提供良好的成长条件。倡导儿童福利社会化，积极探索适合孤残儿童身心发育的供养方式。
　　--加强对流浪儿童救助保护中心的建设和管理。设立多种形式的流浪儿童收容教育机构，减少流浪儿童数量和反复性流浪。
　　--加强正规的残疾儿童康复机构建设，建立健全社区康复和卫生服务机构，对残疾儿童家长进行康复知识培训和指导。

　　组织与实施

　　**一、**国务院妇女儿童工作委员会负责[《纲要》](https://www.pkulaw.com/chl/37733dd7246afab4bdfb.html)的组织实施。国务院各有关部门和社会团体要根据[《纲要》](https://www.pkulaw.com/chl/37733dd7246afab4bdfb.html)的要求并结合各自的职责，制定相应的实施方案。

　　**二、**地方各级政府要结合实际制定本地区儿童发展规划，并纳入当地经济和社会发展总体规划，统一部署，统筹安排。要将[《纲要》](https://www.pkulaw.com/chl/37733dd7246afab4bdfb.html)的实施纳入政府的议事日程，纳入政府主要负责人和主管负责人的政绩考核。

　　**三、**建立健全实施[《纲要》](https://www.pkulaw.com/chl/37733dd7246afab4bdfb.html)的工作机制。国务院各有关部门和社会团体每年要向国务院妇女儿童工作委员会报告实施[《纲要》](https://www.pkulaw.com/chl/37733dd7246afab4bdfb.html)的工作情况。地方各级妇女儿童工作委员会要建立相应的工作制度和报告制度。要加强调查研究，坚持分类指导、示范先行的工作方法，及时掌握有关情况，总结推广经验。要注重对儿童领域的理论研究。

　　**四、**各级政府应根据财力情况，合理安排实施[《纲要》](https://www.pkulaw.com/chl/37733dd7246afab4bdfb.html)所需经费。要多渠道筹集资金，重点扶持贫困地区和少数民族地区儿童事业的发展。

　　监测与评估

　　**一、**对[《纲要》](https://www.pkulaw.com/chl/37733dd7246afab4bdfb.html)的实施情况实行分级监测评估。要加强儿童发展综合统计工作，增设分性别统计指标，建立和完善分性别数据库。做好信息收集、整理、反馈和交流工作，分析儿童发展现状，预测趋势，评估实施效果，为制定规划、科学决策提供依据。

　　**二、**建立国家和省（自治区、直辖市）的儿童状况监测体系，制定切实可行、科学规范的监测评估方案，全面、动态地监测儿童发展状况。[《纲要》](https://www.pkulaw.com/chl/37733dd7246afab4bdfb.html)分性别的统计指标纳入国家统计制度和各有关部门的常规统计和统计调查。建立健全劳动监察、卫生监测、教育督导、统计评估、法律监督机构，完善监测机制，确保[《纲要》](https://www.pkulaw.com/chl/37733dd7246afab4bdfb.html)的有效实施。

　　**三、**建立定期报送和审评制度。国务院妇女儿童工作委员会各成员单位和各有关部门，每年要向国务院妇女儿童工作委员会办公室和国家统计局报送监测数据及目标实施进展情况，并对报送的监测数据进行分析，评估实施效果。国家监测评估周期分为年度监测、每3-5年的阶段性监测评估和10年的终期监测评估。

　　**四、**建立监测评估机构。国务院妇女儿童工作委员会设立监测评估领导小组，负责审批监测评估方案，根据监测评估结果提出相应对策。
　　监测评估领导小组下设统计监测组和专家评估组。
　　统计监测组由国家统计局牵头，相关部门共同组成。负责制定[《纲要》](https://www.pkulaw.com/chl/37733dd7246afab4bdfb.html)分性别的统计监测指标体系，提出监测的重点指标；确定监测方法，收集监测数据，建立和完善分性别数据库；向国务院妇女儿童工作委员会提交全国儿童状况统计监测报告；指导各地区对[《纲要》](https://www.pkulaw.com/chl/37733dd7246afab4bdfb.html)的统计监测工作。
　　专家评估组由国务院妇女儿童工作委员会办公室牵头，相关部门推荐专家组成。负责制定检查评估方案；审评监测报告，并对重点、难点问题的解决提出意见和建议；开展阶段性评估，向国务院妇女儿童工作委员会提交评估报告；指导各地区对实施[《纲要》](https://www.pkulaw.com/chl/37733dd7246afab4bdfb.html)的检查评估工作。
　　各地区要建立相应的监测评估机构和制度，及时、准确、全面地反映[《纲要》](https://www.pkulaw.com/chl/37733dd7246afab4bdfb.html)和本地区规划的实施情况。

© 北大法宝：（[www.pkulaw.com](https://www.pkulaw.com/)) 专业提供法律信息、法学知识和法律软件领域各类解决方案。北大法宝为您提供丰富的参考资料，正式引用法规条文时请与标准文本核对。
欢迎查看所有[产品和服务](https://www.pkulaw.com/helps/2.html)。法宝快讯：[如何快速找到您需要的检索结果？](https://www.pkulaw.com/helps/70.html)[法宝](https://www.pkulaw.com/helps/69.html)V6有何新特色？

扫描二维码阅读原文

**原文链接：**<https://www.pkulaw.com/chl/707cf9cabac6a977bdfb.html>
